# Early childhood bilingualism: effects on brain structure and function

**DOI:** 10.12688/f1000research.23216.2

**Published:** 2020-11-04

**Authors:** Sezgi Goksan, Froso Argyri, Jonathan D. Clayden, Frederique Liegeois, Li Wei

**Affiliations:** 1 Centre for Applied Linguistics, UCL Institute of Education, 20 Bedford Way, London, WC1H 0AL, UK; 2 Developmental Neurosciences Research and Teaching Department, UCL Great Ormond Street Institute of Child Health, 30 Guilford Street, London, WC1N 1EH, UK

**Keywords:** Bilingual, children, brain, MRI, language, executive control.

## Abstract

Growing up in a bilingual environment is becoming increasingly common. Yet, we know little about how this enriched language environment influences the connectivity of children’s brains. Behavioural research in children and adults has shown that bilingualism experience may boost executive control (EC) skills, such as inhibitory control and attention. Moreover, increased structural and functional (resting-state) connectivity in language-related and EC-related brain networks is associated with increased executive control in bilingual adults. However, how bilingualism factors alter brain connectivity early in brain development remains poorly understood.

We will combine standardised tests of attention with structural and resting-state functional magnetic resonance imaging (MRI) in bilingual children. This study will allow us to address an important field of inquiry within linguistics and developmental cognitive neuroscience by examining the following questions: Does bilingual experience modulate connectivity in language-related and EC-related networks in children? Do differences in resting-state brain connectivity correlate with differences in EC skills (specifically attention skills)? How do bilingualism-related factors, such as age of exposure to two languages, language usage and proficiency, modulate brain connectivity?

We will collect structural and functional MRI, and quantitative measures of EC and language skills from two groups of English-Greek bilingual children - 20 simultaneous bilinguals (exposure to both languages from birth) and 20 successive bilinguals (exposure to English between the ages of 3 and 5 years) - and 20 English monolingual children, 8-10 years old. We will compare connectivity measures and attention skills between monolinguals and bilinguals to examine the effects of bilingual exposure. We will also examine to what extent bilingualism factors predict brain connectivity in EC and language networks.

Overall, we hypothesize that connectivity and EC will be enhanced in bilingual children compared to monolingual children, and each outcome will be modulated by age of exposure to two languages and by bilingual language usage.

## Introduction

Early life experiences shape brain and cognitive development ^1, 2^. The study of bilingualism provides a unique model to examine the neural changes linked to early experiences, i.e. whether there are early bilingual language experience effects on the brain, since two languages can be acquired from birth or one language can be acquired from birth and a second language later in life ^3– 5^. Despite there being a greater number of multilingual adults in Europe compared with monolinguals ^6^, the effect of early bilingual language exposure on brain structure and function is still poorly understood ^4^. Crucially, most research has focused on adults (see 7 for a review), after decades of bilingualism exposure, alongside other experiences, making it increasingly difficult to associate changes in brain networks with specific experience-related factors.

Our study addresses the following question: Are brain connectivity changes in bilingual children mainly driven by (i) brain maturation stage at age of exposure to two languages, or by (ii) experience-related factors, for example, frequency of use of both languages?

A wide body of research suggests that bilingualism confers advantages in executive control (EC), i.e. the ability to control attention, to inhibit distractions and to shift between goals (e.g. 8, but see 9 for an opposing view). It is hypothesized that this “bilingual advantage” is linked to the ongoing need to manage two language systems, for which EC is required ^3^. Brain networks involved in supporting or engaging EC include the fronto-parietal control network (FCN), salience network (SLN) and default mode network (DMN) ^10^, used for attending, switching/inhibitory control and disengaging in response to external stimuli, respectively ^11– 14^. In short, early bilingualism in combination with regular practice of two languages may be associated with better performance on some EC tasks, as well as increased connectivity between EC-related brain networks, because of increased time spent using and controlling two language systems ^15, 16^.

Although there are a growing number of functional magnetic resonance imaging (MRI) studies investigating the impact of bilingualism on brain activity during executive function tasks (for example 17, 18, in adults and 19 in children), there are very few studies on the effects of bilingualism on resting brain networks known to be related to executive function ^20^. Specifically, few studies have asked whether bilingual experience modifies resting-state brain networks, such as (i) the FCN (which includes dorsolateral frontal regions and the inferior/superior parietal lobules), (ii) the SLN (which includes the anterior insula and the dorsal anterior cingulate gyrus), and (iii) the DMN (which includes the posterior cingulate gyrus, the ventromedial prefrontal cortex (vmPFC) and the inferior parietal lobule/angular gyri) ^10, 20– 27^. An investigation addressing this question in bilingual adults found there was greater negative correlation between the vmPFC (part of the DMN) and the dorsolateral prefrontal cortex (part of FCN) in simultaneous vs. sequential bilinguals ^27^. Moreover the stronger this interaction, the quicker bilinguals responded during “interference suppression” trials in a cognitive control (i.e. Simon) task ^27^. Of note, no monolingual adult control group was included in the study. Our study will allow us to disentangle brain alterations in domain-general (EC) vs. in language-specific networks in bilingual and monolingual children.

Additionally, bilingualism factors such as age of exposure to two languages, language usage and proficiency have been shown to impact differences in functional brain activity and connectivity ^28, 29^. One study has reported that earlier age of acquisition of two languages was associated with stronger functional connectivity between the left inferior frontal gyrus pars triangularis (IFGpt, i.e. Broca’s area, BA 45) and its right homolog and with the right inferior parietal lobule (IPL, part of the FCN) ^4^. A more recent study also found the same relationship in the bilateral IFG in a different group of highly proficient bilingual adults ^30^. Furthermore, the authors observed that greater ‘diversity of language use’ (i.e. an environment in which both languages are commonly utilised and segregated use of each language is not routine) was positively correlated with functional connectivity between cortical and subcortical brain regions (namely between the anterior cingulate cortex and left putamen, and the left caudate and bilateral superior temporal gyrus (STG)) ^30^. Altogether, there is emerging evidence that bilingualism factors influence brain functional connectivity, yet little is known about how these connectivity alterations are related to EC performance. 

There is also growing evidence that bilingualism alters the grey and white matter structure in the brain. For example, in adult studies, greater volume and grey matter density are found in regions associated with language, such as the bilateral IFG, IPL, anterior cingulate cortex, caudate and putamen ^31– 35^ However, a recent study in pre-school children (aged between 3 and 5 years) found evidence of greater functional connectivity in bilinguals than monolinguals but no structural differences in the IFG, thus suggesting that structural changes may only appear after prolonged exposure to two languages ^36^.

Diffusion-weighted MRI studies have revealed white matter alterations associated with bilingualism (for example, see 37 for a recent review), as measured by indices related to structural connectivity such as Fractional Anisotropy (FA) ^38^. Increased FA in language-specific white matter tracts, such as the inferior fronto-occipital fasciculus (IFOF) has been reported in both adults ^39^ and children (aged between 8 and 11 years old) ^40^. Mohades and colleagues (2012) showed that simultaneous bilingual children (who had exposure to both language before 3 years old) had greater FA within the IFOF compared to successive bilingual children (where age of exposure to two languages was between 3 and 5 years) ^40^. As most MRI studies have focused on adults, it is difficult to disentangle the effects of early environmental changes from those of decades of exposure to bilingualism and other experiences.

In order to disentangle the effects of bilingualism factors and maturational factors on connectivity, we will recruit bilingual children with either early or later (3 to 5 years) age of onset of exposure to two languages and extensively characterise their linguistic experience.

The effects of bilingualism on brain structural and functional connectivity can be observed as soon as most EC skills are well developed. We will recruit bilingual children aged 8 – 10 years old because this is before the time of significant brain changes related to puberty, yet an age when EC skills are well developed ^41^.

## Objectives

Our overall aim is to investigate whether bilingual experience alters EC- and language-related brain networks in children using advanced MRI methods.

Specifically, we will

(i) examine whether structural and functional connectivity at rest (‘connectivity’ hereafter) differs between two groups of bilingual children and one group of monolingual children;
(ii) identify how age of onset of exposure to two languages, language usage, and proficiency in two languages modulates brain connectivity.

### Hypotheses and predictions

(1) *Bilinguals vs. Monolinguals.* Early bilingualism will affect connectivity in specific executive control (EC) networks that have been implicated in the ability to engage EC. Both bilingual groups will have stronger functional connectivity within the FCN and DMN networks than monolinguals (as seen for adults in 10).
(2) *Simultaneous vs. Successive Bilinguals.* The effect of bilingualism on language and EC connectivity will be moderated by age of onset of exposure to two languages. Specifically, the bilingual simultaneous group will show enhanced FCN connectivity relative to the successive bilingual group (as seen for adults in 4).
(3) Within the bilingual groups, language usage and proficiency will moderate the effect of bilingualism on (i) the language network and (ii) the EC network involved in inhibition, i.e. the SLN ^14^, as seen behaviourally ^15^. Therefore, we predict a positive correlation between proficiency/usage of both languages and connectivity within the SLN network, and within the language network, across bilingual participants.

## Protocol

### Participants

***Inclusion criteria.*** Three groups of typically developing monolingual and bilingual children aged between 8 and 10 years of age will be recruited.

**Group 1:** English monolingual children, who have exclusively been exposed to English at home since birth (i.e. they are born to English-speaking parents).

**Group 2:** English-Greek simultaneous bilingual children, exposed to these two languages from birth.

**Group 3:** English-Greek successive bilinguals, where exposure to Greek was from birth and exposure to English was between the ages of 3 and 5 years (children are born in Greece and arrived in the UK between the ages of 3 and 5 years).

***Exclusion criteria.*** Children are excluded from the study if they (a) have had regular exposure from a young age to other languages other than English and Greek; (b) if their Greek-speaking parents were born and/or have lived in the UK for most of their lives, and did not migrate from Greece to live in the UK during adulthood; (c) are not in mainstream schooling; (d) have any contraindications for MRI (e.g. have metal implants or braces); (e) have history of hearing impairment and/or have been diagnosed with language learning difficulties; (f) have a confirmed or suspected diagnosis of developmental conditions (e.g. ADHD, autism, dyslexia), neurological conditions (e.g. epilepsy, cerebral palsy), severe chronic medical condition or being born extremely premature (earlier than 33 weeks gestational age); or (g) have non-verbal intelligence below 70 (assessed using Raven’s Coloured Progressive Matrices, see Methodology section for more detail).

### Ethical approval

The research project has been approved by the UCL Institute of Education Research Ethics Committee (approval number: 1080). Prior to participation, researchers will obtain written informed consent from one parent/guardian and written informed assent from each child participant.

### Methodology

***Procedure.*** Standardised tests are used to measure the children’s linguistic and non-linguistic abilities. The bilingual children’s parents are also asked to complete a questionnaire recording detailed information about their child’s exposure to each language. Furthermore, all children are invited to take part in an MRI brain scanning session during which structural (anatomical and diffusion-weighted) and functional (resting-state) images are acquired.

***Background measures: non-verbal ability and verbal working memory.*** The Raven’s Coloured Progressive Matrices (CPM) test is used to assess the non-verbal ability of the child participants (suitable for use with children aged 4 to 11 years ^42^). During the task each child is asked to complete a puzzle by choosing the correct missing piece from six options. A total of 36 puzzles are presented so that each participant can obtain a maximum score of 36 (one point for each puzzle). Raw CPM scores are then converted to an age-appropriate normalised score (standard score). Standard scores have mean = 100 and standard deviation (SD) = 15 ^42^. The lowest and highest scores a child can get are <60 or >140 respectively. No significant gender differences were found in a large, normative sample reported by Raven et al., and distributed by Pearson Inc. (Girls: mean = 101.44, SD = 15.74, Boys: mean = 99.31, SD = 15.2 ^42^).

Verbal working memory is assessed using subtests that involve repeating series of numbers forwards and backwards (CELF-4 UK ^43^). The use of this task is advantageous because it involves minimal linguistic/lexical demand, as children only need to be familiar with digits 1 to 9. It is also suitable for children aged between 5 and 16 years old. Sequences are presented until the child fails to recall two consecutive sequences. The maximum possible raw score for each task is 16 and 14. Standard scores range from 1 to 19 (mean = 10, standard deviation = 3, CELF-4 UK ^43^).

Fluid intelligence, working memory and controlled attention are related yet separable constructs ^44– 46^. We have therefore chosen to collect measures of non-verbal fluid intelligence and verbal working memory as background variables, as in previous studies that have investigated executive control skills in bilingual children and adults ^47– 51^. Based on Engle *et al*.’s theory, individual differences in working memory capacity and general intelligence will impact a child’s innate ability to control their attention ^45^. Moreover, it has been observed that greater resting-state connectivity of the precuneus/posterior cingulate with other regions of the default mode network, is positively correlated with working memory performance in adults ^52^. Therefore, we aim to account for individual variability related to brain connectivity by including scores for working memory and general intelligence as confounding factors in our connectivity analyses.

***Expressive vocabulary.*** The Raven’s Crichton Vocabulary Scales (CVS) test is used to assess verbal ability (suitable for use with children aged 4 to 11 years ^42^). The CVS is a standardised expressive vocabulary test designed for use with the CPM. It assesses the knowledge of words and is constructed to cover as closely as possible the same age range of intellectual development as the CPM. During the CVS, children are asked to describe the meaning of words in a list (80 words). Both English and Greek vocabulary are assessed for bilinguals and only in English for monolinguals. Total raw vocabulary scores are then converted to standard scores (mean = 100, SD = 15). No significant gender differences were found within two large, normative samples, distributed by Pearson Inc. (English – Girls: mean = 101.05, SD = 15.14, Boys: mean = 99.92, SD = 15.47; Greek – Girls: mean = 66.5, SD = 43.6, Boys: mean = 67.0, SD = 41.8 ^42^).

***Executive control.*** We also record performance on EC skills, focusing on the selective, sustained and switching components of attention. These are assessed using four subtests from a standardized computer-based test (TEA-Ch 2 ^53^).

(1) Attention switching is measured in a subtest that involves sorting four objects according to two defined categories (bags and shoes are sorted for either their colour or whether they pair with a hand or feet).
(2) Selective attention is measured via a subtest of target detection amongst distractors.

Sustained attention is measured by the final two tasks;

(3) Children have to detect target sounds (dog barks) while ignoring distractors (other animal noises) and they must concentrate in order to do this successfully for 15 trials (the final score is weighted for accuracy on all trials).
(4) Each child’s reaction time (RT) is measured during a 5 minute test (pressing a button in response to the appearance of a blue blob on the screen).

The composite scores per subtest are calculated as follows: (1) mean RT for switch trials (only correct responses used to calculate the mean), (2) average number of correct responses in two 30 s trials, (3) mean RT weighted for accuracy, and (4) mean RT. The computer-based TEA-Ch 2 assessment produces a PDF output with composite scores and standard scores (either population-based or education-referenced; mean = 10, standard deviation = 3) for each subtest. Standard scores range from 1 – 19.

Inhibitory control in the form of inhibition of a prepotent response is likely to be invoked during the switching attention task. During this task, participants must ignore goal-irrelevant stimuli (for example whether the object is red or blue) in order to produce a goal-relevant response (i.e. whether the object pairs with a hand or feet). It is of note that the rules in this task are presented in blocks of 5 and the mean reaction time is measured as the average response to the ‘switch trials’ only, i.e. the first response in each block where the rule has switched from either a red/blue decision to a hand/feet decision or vice versa. 

***Parental questionnaire on history of language exposure and biographical information.*** Detailed information on the bilingual children’s exposure to the two languages is collected via a structured parental questionnaire (ALEQ Heritage developed by Daskalaki *et al.,* 2019 ^54^, based on the Alberta Language Environment Questionnaire (ALEQ) ^55^). The questionnaire includes questions about family demographics, age and length of exposure to the two languages, the child’s and the parents’ place of birth, language use among the bilingual child and family members in the home, other contexts of bilingual exposure and use (e.g. extracurricular and literacy activities), time of relocation to the UK, and parents’ education levels. Information about the socioeconomic status of the family will be calculated based on years of maternal education.

The original ALEQ was designed to measure the current English language use (input and output) in the bilingual child’s environment. The adaptation we use (ALEQ Heritage), however, measures the current Greek language use, and thus the 5-point Likert rating scale has been adapted accordingly. For each question in ALEQ Heritage, the scale is as follows: 0-English always/Greek never, 1-English usually/Greek seldom, 2-English 50 %/Greek 50 %, 3-English seldom/Greek usually, 4-English never/Greek always.

For the purposes of the current study we will extract the following outcome measure(s):

*Language use at home*

Language use at home is calculated by taking the average of two values: (i) Greek/English input and (ii) Greek/English output. Input and output are generated by inserting the Likert scale scores (in response to selected questions) in to the following formula: ( *sum of scores*) / ( *number of scores x* 4) (see page 7 of Paradis’ ALEQ). Input is measured using questions about how frequently family members speak Greek/English to the child using the 5-point Likert scale discussed above, i.e. from 0 (English always/Greek never) to 4 (English never/Greek always). Output is measured using questions that explore the frequency with which the child speaks Greek/English to family members at home (on the same 0 - 4 scale). Greek input and output scores are then averaged to produce a score for language use at home, ranging from 0 to 1. This score provides a quantitative measure of the amount of English/Greek input and output the child receives and directs to family members. Higher language use scores (> 0.5) indicate a higher relative use of Greek language at home, whereas lower scores (< 0.5) indicate a higher relative use of English.

*Richness score*

English and Greek richness scores range from 0 to 1. Each score is a proportion (out of 32) representing English/Greek language use during a range of activities both inside and outside the home. Activities include how frequently English and Greek are used with friends, during literacy activities (e.g. reading books, online language games, etc), and during other extra-curricular activities (e.g. music lessons, sports, etc) on a weekly basis. The calculation of richness scores in the adapted version of the questionnaire that we use differs from the original ALEQ because it includes more questions. The key differences are highlighted here. Firstly, Questions 28 and 31a have been combined and the focus is on how much formal Greek education the child receives. This combined question is included in the calculation of both English and Greek richness scores (rather than only being included for the ‘mother tongue’ in the original ALEQ, thus adding 4 extra points to the English score). In question 30 more activities have been added such as ‘use of Skype’, ‘use of mobile phone’, therefore Greek and English each have a maximum score of 14. In addition, question 32 has been repeated and can be included in the final richness score two further times (4 points per question). This captures more contexts in which children socialize with friends or relatives. Therefore, the total denominator is currently 32 for both English and Greek vs. 16 and 20 respectively in the original ALEQ, (see page 10 of Paradis’ ALEQ).

*Combined Greek score*

A combined score will be calculated and used to denote Greek language use (range 0 – 2). The score will be generated by adding together each child’s scores for language use at home and Greek richness. The combined score will more accurately represent each child’s exposure to and use of Greek in various contexts both inside and outside the home.

***Magnetic Resonance Imaging (MRI) scans.*** Children are scanned on a 3T Magnetom Prisma scanner (Siemens). The MRI scanner is within a child-friendly environment with images of fish on the walls. The scanner room is also equipped with a television screen that can be used to play a movie for children during the acquisition of structural images.

To prepare children for the scanner environment we use a booklet designed for children having an MRI scan at Great Ormond Street Hospital. In addition we create a mock scanning session before each scan where children practice laying still in a toy fabric tunnel while listening to scanner noises. Children can also choose whether to have their parent accompany them and remain in the scanner room for the duration of the scan. Each scanning session lasts approximately 45 minutes. This includes the time taken to set up and settle each participant followed by collection of the structural, diffusion and functional resting state images (total acquisition time approximately 22 min).

Data are acquired using a 64 channel head coil. A high-resolution magnetisation prepared rapid gradient echo (MPRAGE) T1-weighted 3D image is acquired for anatomical reference per participant using the following parameters (repetition time (TR) = 2300 ms, echo time (TE) = 2.74 ms, inversion time = 909 ms, flip angle (FA) = 8°, acquisition time (TA) = 5 min 21 s, field of view (FOV) = 256 × 256 mm, matrix size = 256 × 256, 240 slices, 1 mm isotropic voxels, single shot, slice acquisition = ascending, parallel acquisition technique (PAT) = GRAPPA). 3D diffusion-weighted MRI scans are collected using a single-shot multi-shell diffusion MRI sequence. The images are encoded along 60 independent directions with b-values of 1000 and 2200 s/mm ^2^ (TR = 3050 ms, TE = 60 ms, FA = 90°, TA = 7 min 16 s, FOV = 220 × 220 mm, matrix size = 110 × 110, voxel size = 2 × 2 × 2 mm, number of contiguous axial slices = 66, slice thickness = 2mm, distance factor = 10 %, slice acquisition = interleaved, PAT = GRAPPA, multi-band acceleration factor = 2, fat suppression = on). We also acquire 13 b0 images and one reverse phase encoded b0.

Resting-state functional MRI (rs-fMRI) are acquired at the end of the scanning protocol, so children have become comfortable with the scanning environment. During the rs-fMRI scan children are asked to fixate their gaze on a cross. This method has been shown to increase reliability in connectivity metrics ^56^. The acquisition parameters for the resting scan are as follows: T2* BOLD-weighted, GRE, EPI readout, FA = 75°, TE = 26 ms, TR = 1240 ms, TA = 6 min 18 s, number of measurements = 300, FOV = 200 mm, multi-band 2, 80×80 in-plane matrix size, 2.5 mm isotropic voxels, 40 slices, slice thickness = 2.5 mm, distance factor = 20 %, slice acquisition = interleaved, fat suppression = on, and with a single-band reference (SBref) image acquired at the start. A field map is also acquired (GRE, 2D, dual echo TE1/TE2 = 10/12.46 ms, TR = 1020 ms, TA = 2 min 47 s, FA = 90°, FOV = 200 mm, matrix size = 80 × 80, voxel size = 2.5 × 2.5 mm, 40 slices, slice thickness = 2.5 mm, distance factor = 20 %, slice acquisition = interleaved).

### MRI data analysis

**Figure 1.**
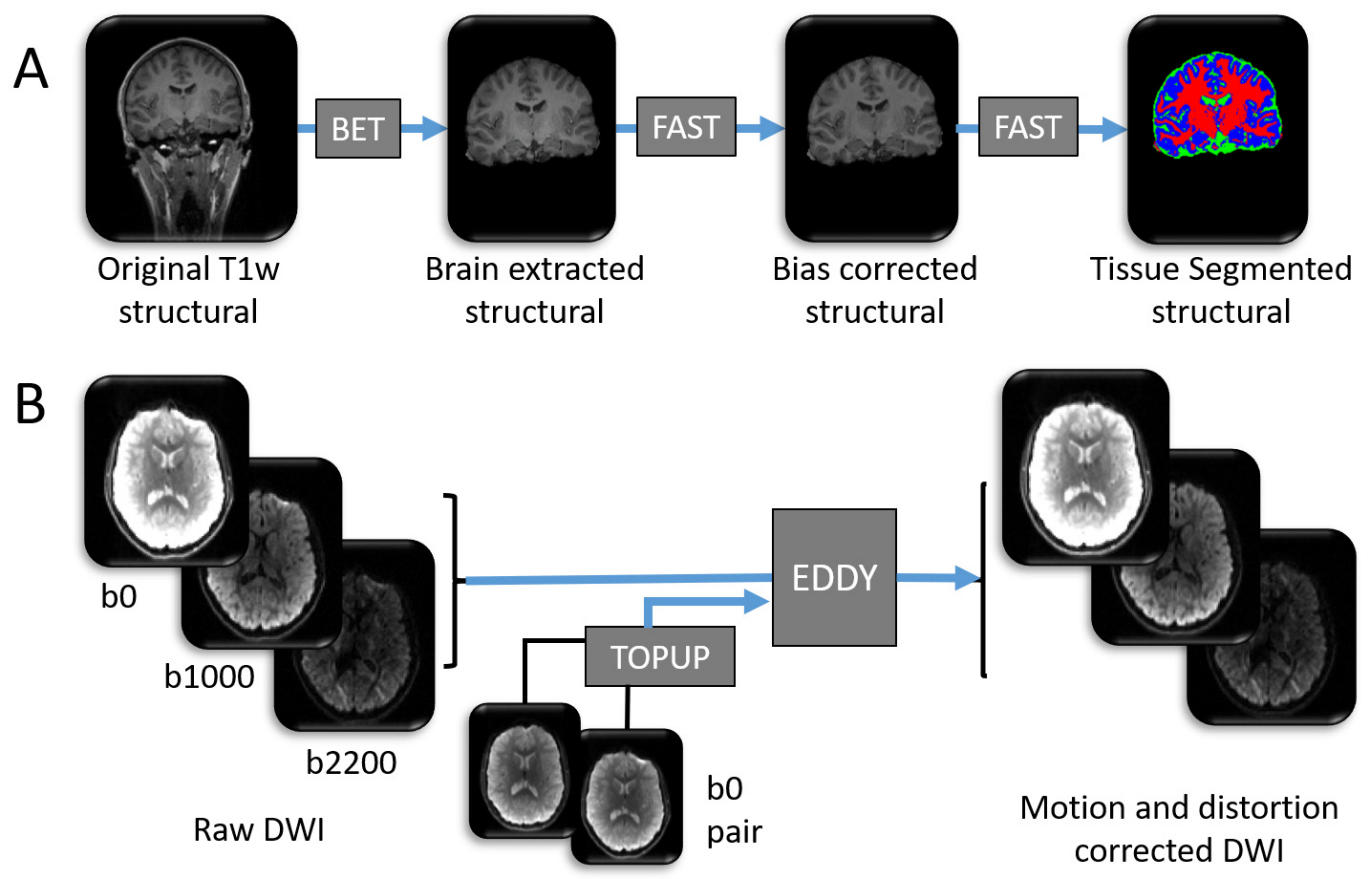
Graphical representation of the structural MRI data analysis pipelines. A summary of the data processing tools used to prepare the ( **A**) T1 weighted image and ( **B**) diffusion weighted image for further analysis. Blue arrows denote pre-processing steps using freely available online tools (named within dark grey boxes). All pictures are of 3 dimensional images from one participant, and represent examples of input and output data files. As part of process ( **A**), each high resolution T1 weighted structural image has (i) non-brain tissue removed using BET, (ii) spatial intensity variations (RF field inhomogeneity) corrected using FAST and (iii) brain tissue types automatically labelled to one of either grey matter, white matter or cerebrospinal fluid (CSF) using FAST. Blue = grey matter, red = white matter, green = CSF. High resolution structural images are shown in greyscale. Note: this figure does not contain all input files and options required for each tool. For a comprehensive list of inputs, see the scripts that accompany this analysis ^70^.

**Figure 2.**
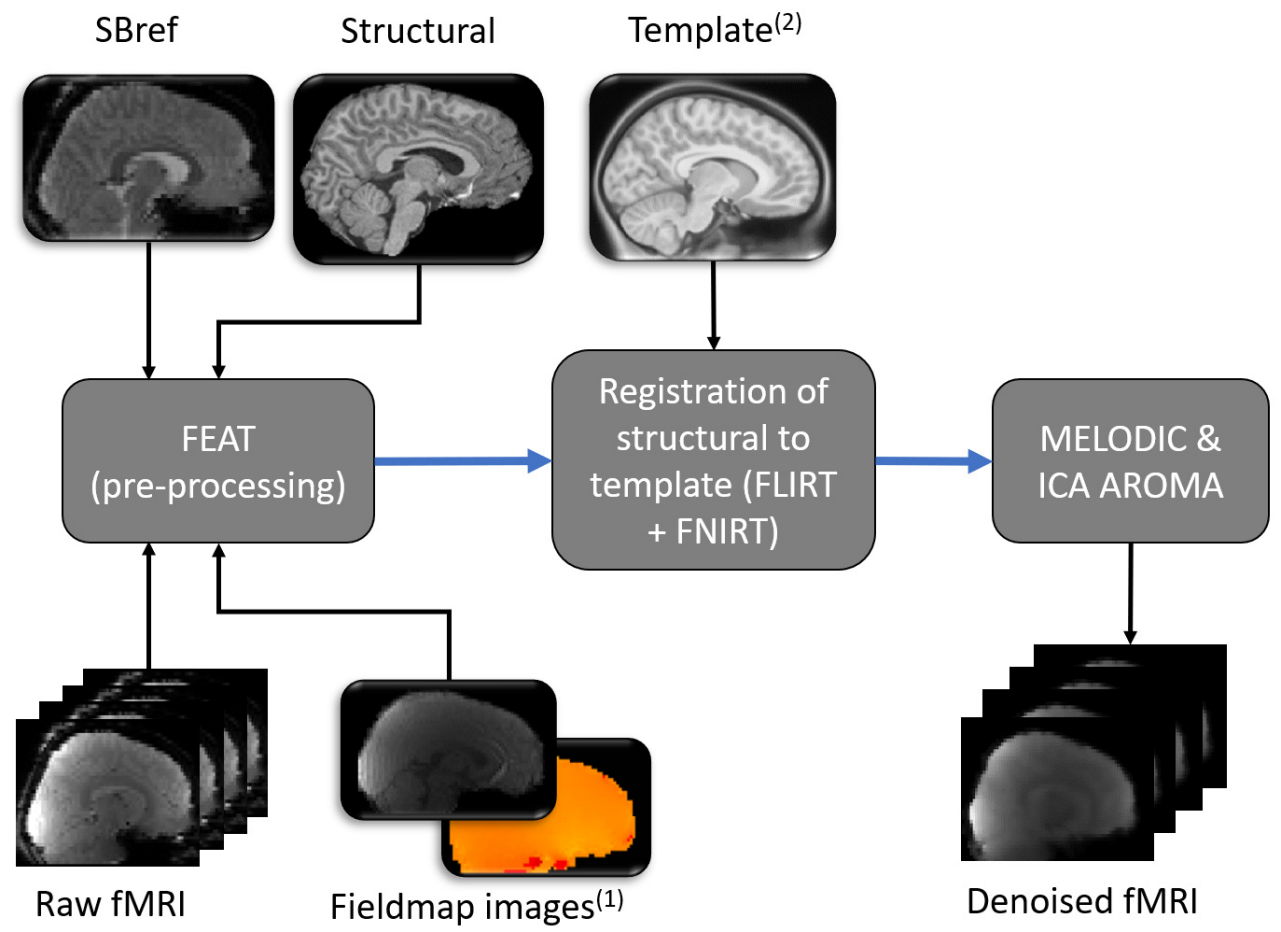
Graphical representation of functional MRI analysis pipeline. Images from one participant represent examples of input and output data files. Blue arrows denote pre-processing steps using freely available online tools (named within dark grey boxes). Pictures with round corners represent 3 dimensional images, while a series of pictures represents 4 dimensional functional data. FEAT was run using the graphical user interface (rather than by running a script), therefore additional required options are detailed within the main text. Subscript numbers highlight steps that were modified in relation to a typical adult pipeline (see Figure 3 for (1) and Figure 4 for (2)).

All data are pseudonymised. High-resolution MRI data has visibly identifiable features of the face removed. Data collected from paediatric participants often requires additional, bespoke consideration; in particular, such data can often contain more motion effects than normal. Therefore, all brain imaging data have visual quality checks to assess the presence of motion artefacts. For the structural and functional data automated quality control descriptors are generated (MRI quality control (MRIQC ^57^)). For the diffusion weighted images, eddy quality control is used (eddy_qc ^58^). The resulting descriptors will be used to detect excessive motion in relation to the rest of the data. Specific pre-processing pipelines (i.e. a series of analysis steps) are subsequently implemented for the structural, diffusion and resting-state functional MRI images (see Figure 1 and Figure 2, and see https://github.com/sgoksan/paed_mri_preprocessing for detailed code).

**Figure 3.**
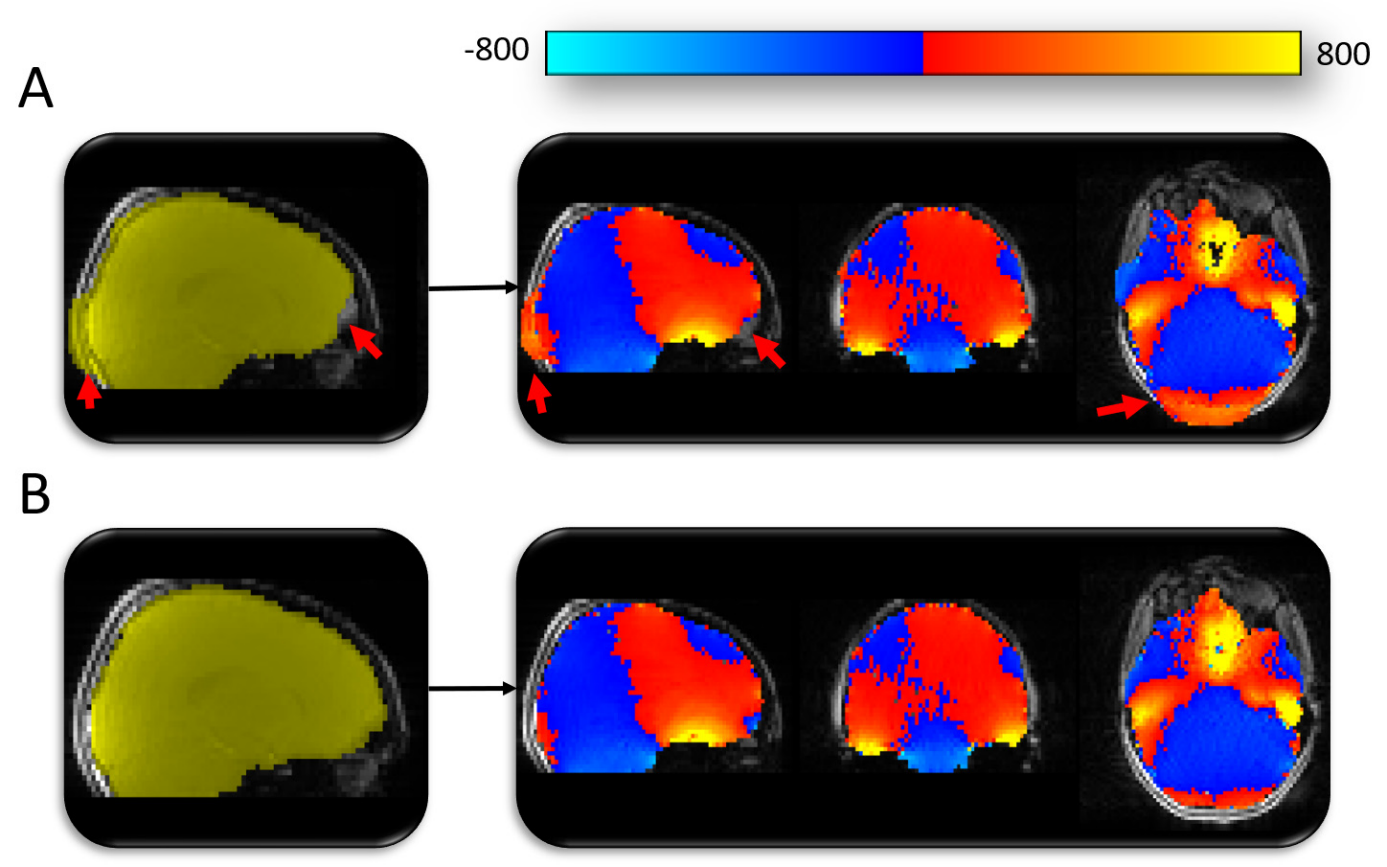
Manual editing of masks for creating fieldmaps. ( **A**) Automated mask generated using BET (shown in yellow). The automated methods used to mask the fieldmap magnitude image were producing sub-optimal results (highlighted by red arrows). Fieldmap image (blue-red-yellow image) generated using the sub-optimal mask would leave distortion-susceptible brain regions without appropriate distortion correction (e.g. red arrow in frontal lobe region). ( **B**) Mask following manual editing (yellow) and the subsequent fieldmap (blue-red-yellow) with appropriate coverage of distortion-susceptible regions.

**Figure 4.**
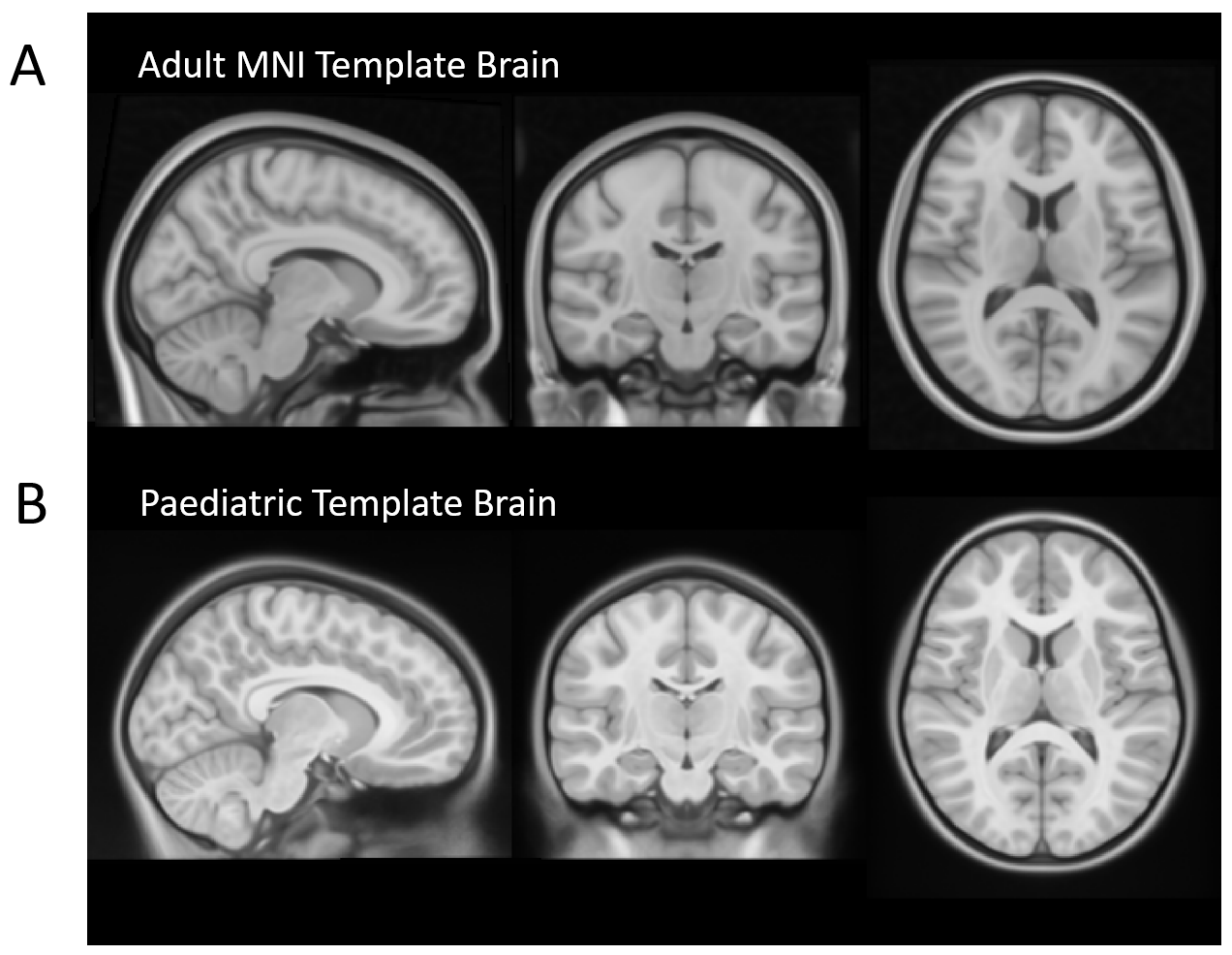
Adult MNI compared with the NIH Paediatric Template Brain. ( **A**) Adult 1mm MNI Template Brain which is commonly used as the standard space image for functional MRI analysis (available as part of FSL). ( **B**) A symmetric paediatric template brain that has been created by Fonov *et al.*, 2011 ^59^, using structural images from 112 children, aged 7 – 11 years. Use of an age-appropriate template is important in paediatric studies due to important differences between the brains of adults and children. For example, children have less developed frontal lobes, thinner corpus callosum and smaller ventricles.

As most software has been developed for use with adult data, some aspects of this analysis pipeline have been tailored for a paediatric cohort. These steps included (1) manual editing of fieldmap masks (see Figure 2 and Figure 3), (2) use of a paediatric template brain for registration of structural images (left-right symmetric, created by Fonov *et al.,* using structural images from 112 children, aged 7 – 11 years ^59^, see Figure 2 and Figure 4), (3) modification of inputs to ICA-AROMA in order to accept a paediatric template and related masks.

MRI data will be analysed using a combination of MRI analysis tools within FMRIB Software Library (FSL) version 6.0.2 ^60– 62^ and MRTrix3 ^63^.

***Structural T1.*** Initial steps on the structural T1-weighted image include brain extraction (i.e. removal of non-brain tissue from the image) using FMRIB’s Brain Extraction Tool (BET ^64, 65^), correction of RF inhomogeneity (spatial intensity variations), and segmentation of the different brain tissue types in the T1-weighted structural images using FMRIB’s Automated Segmentation Tool (FAST ^66^) (see Figure 1A).

***Diffusion weighted data.*** For the diffusion weighted structural images, corrections for susceptibility-induced distortions, eddy currents and movements of the head are performed using TOPUP and EDDY (part of FSL) ^60, 67– 69^ (see Figure 1B).

In addition, given our paediatric cohort, the final set of diffusion images will be carefully assessed to establish whether further denoising and removal of Gibbs ringing artefacts are required. These steps have been recommended for use in adult studies ^71^, and are available steps within MRTrix3 ^72, 73^.

**Figure 5.**
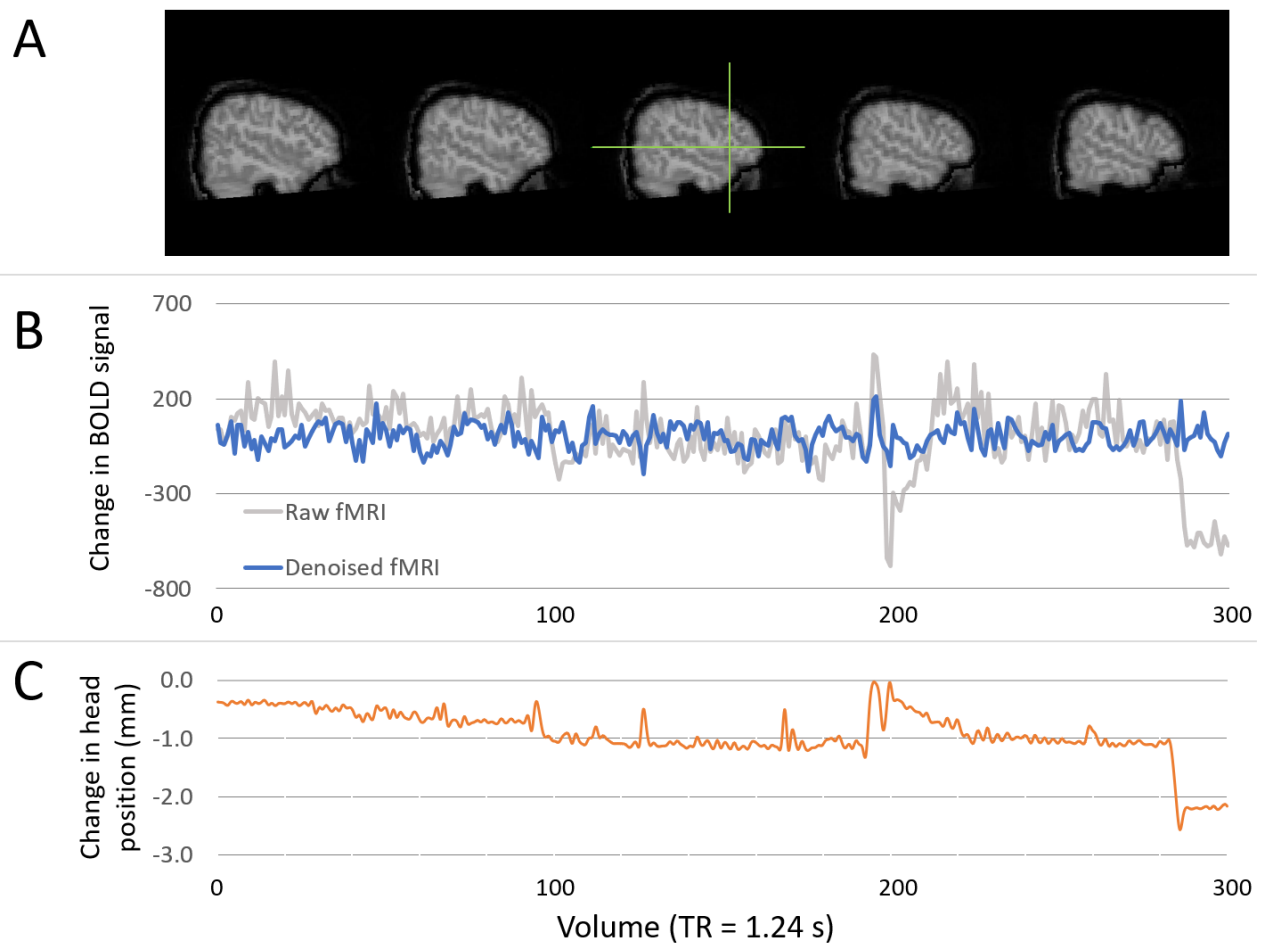
Example of denoising functional data. ( **A**) Images show the location of a chosen voxel (green crosshair), within the left inferior frontal gyrus (pars triangularis). ( **B**) Time series plots showing the change in BOLD signal within the specified voxel for raw data (grey line) and final denoised and temporal filtered data (blue line). All time series are demeaned. Units of change in BOLD are arbitrary scanner units. ( **C**) Plot showing the change in head position (left-right) in millimetres. Changes in head position align with large changes in BOLD signal, which are successfully minimised through denoising (via the use of ICA-AROMA).

**Figure 6.**
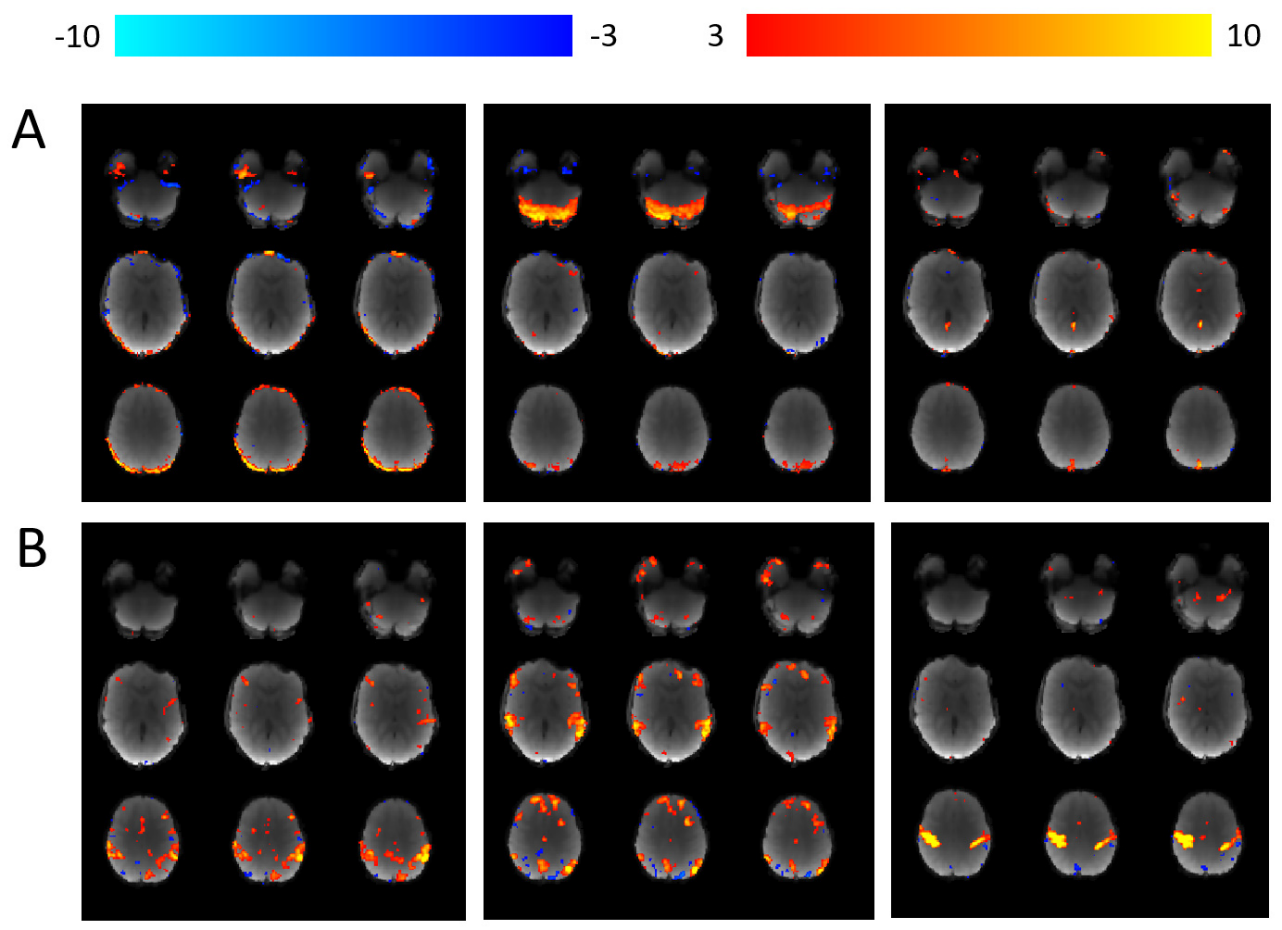
Independent components within a single participant’s resting-state fMRI data. Using MELODIC ^79^, each individual participant’s 4 dimensional fMRI data is decomposed in to independent spatial and temporal components. ICA-AROMA is then used to (i) automatically identify “movement” components and (ii) remove them from the data ^80^. ( **A**) Examples of three separate components labelled as “movement” and subsequently removed from the data. ( **B**) Examples of three separate components that were not labelled as movement and therefore remain within the data.

***Resting-state functional data.*** For the functional resting state data, FEAT (Version 6.00 ^74^) will be used to run motion correction of the functional data using MCFLIRT ^75^, distortion correction using FUGUE, brain extraction using BET ^76^ and grand mean scaling ^74^. The following images will be prepared for input into FEAT: (1) reference image for motion correction of the functional data and (2) fieldmap and fieldmap magnitude images. A single-band reference (SBref, acquired at the start of the functional scan) will be bias corrected using FAST and then used as an alternative reference image for motion correction. The fieldmap magnitude image will initially have non-brain matter removed using BET and will then be manually edited (see Figure 3). Subsequently, the fsl_prepare_fieldmap function will be used to create a fieldmap image (for an example see Figure 3). FEAT will also register the SBref to the T1 weighted structural image using FMRIB’s linear image registration tool (FLIRT, rigid-body and boundary-based registration) ^75, 77, 78^. FLIRT and FSL’s non-linear image registration tool (FNIRT) are then used to register each participant’s structural T1 weighted image to a Paediatric Template image ^62^ (see Figure 4 for example of Paediatric Template). MELODIC (model-free fMRI analysis using probabilistic independent component analysis) will decompose functional data into spatially independent components ^79^, which will subsequently automatically be labelled as signal (i.e. not movement) or noise (i.e. motion or physiological artefact) using ICA-AROMA (Automatic Removal of Motion Artifacts ^80^). ICA components that depict physiological noise or movement are automatically removed (see Figure 5 and Figure 6). Lastly the data is high pass temporal filtered at 0.01 Hz (100 s period).

### Statistical plan

We will compare the brain structural (mean fractional anisotrophy (FA) from DWI-derived tracts of interest) and functional (mean correlation) connectivity in specific networks involved in language and executive control across our three study groups (i.e. simultaneous bilingual, sequential bilingual and monolingual). We will investigate the specificity of differences by also comparing connectivity indices within two networks not hypothesized to be influenced by bilingualism (visual and motor).

***Tractography reconstruction.*** Diffusion data will be prepared for tractography analysis by estimating fibre orientations using multi-shell, multi-tissue, constrained spherical deconvolution ^81– 83^. Fibre orientation distributions will then be used to calculate mean FA per voxel, as well as run anatomically constrained tractography (ACT), generating streamlines for each tract of interest ^84^. The mean FA per tract (weighted by the number of streamlines in each voxel) will then be extracted per participant. The five tracts of interest are two pathways involved in language, one involved in executive control and two control pathways: (a) arcuate fasciculus (dorsal language pathway), (b) inferior fronto-occipital fasciculus (ventral language pathway), (c) fronto-parietal tract (d) optic radiation tract (e) corticospinal tract.

***Resting-state network connectivity.*** In order to investigate functional connectivity, we will extract mean blood oxygen level dependent (BOLD) responses from regions of interest (ROI) in selected resting-state networks. The BOLD responses (i.e. measurements of changes in BOLD signal collected every 1.24 s for the duration of the functional scan) will be calculated from each pre-defined region of interest (ROI), detailed below. Measurements are collected per voxel and will be averaged across the ROI to create a mean BOLD response across time. Each mean response will be correlated with those within the same network to produce a network-specific correlation matrix per participant. The bilateral ROIs for each network will be as follows:

(a) language network: the IFG and posterior STG
(b) executive control networks:
  (i) FCN: middle frontal gyrus (part of the dorsolateral prefrontal cortex) and inferior/superior parietal lobule,
  (ii) SLN: anterior insula and anterior cingulate gyrus,
  (iii) DMN: posterior cingulate gyrus, vmPFC, and inferior parietal lobule/angular gyrus,
  (iv) subcortical control regions: putamen and caudate,
(c) visual network: primary visual cortex and lateral geniculate nucleus in the thalamus
(d) motor network: the bilateral precentral gyrus (primary motor cortex region) and ventral lateral thalamic nucleus for functional connectivity.

We will use the following statistical methods to test our hypotheses.

(1) To test whether early bilingualism will affect connectivity in EC networks, we will use graph analysis to calculate the global network efficiency within EC networks and compare this across the three groups.
(2) To test whether age of exposure to two languages influences the mean functional connectivity within language and EC-related networks, we will calculate the average of all absolute connectivity values across all the edges within each network and compare this between the three groups using analyses of covariance.
(3) To test whether age of exposure to two languages, Greek language usage and proficiency influences connectivity within language and EC-related networks we will compare the two bilingual groups on both behavioural measures of attention and of brain connectivity (i.e. mean FA and mean functional connectivity) within EC networks. We will use general linear models to examine how much unique variance in connectivity indices is explained by age of exposure to two languages, Greek language usage, and proficiency.

We will examine our data for effects related to gender, socio-economic status and gestational age at birth. We will subsequently make an informed decision regarding whether to include these factors as covariates in our connectivity analyses. Moreover, all analyses will include measures of brain size (total cortical volume) or non-verbal ability (CPM scores), working memory (scaled score for total forward and backward digit span) and age at MRI as covariates, where appropriate.

All software that will be used for analysis of the data is open access and therefore freely available online.

## Plans for dissemination 

Project findings will initially be disseminated as an open access preprint publication. This will be followed by publication as an original research article in a peer-reviewed journal. We will also share key findings and their implications with educators, policy makers and the wider public. Finally, dissemination to non-academic audiences will be done via public engagement events to schools and parents’ networks through UCL BiLingo (Dr Froso Argyri and Prof Li Wei are Co-founders of this service).

Permission is requested from each parent/guardian in order to make their child’s anonymised data available online. Where open MRI data is a requirement for publication we will make consented, anonymised data available at that time, otherwise we aim to publish the anonymised MRI imaging data (for which permission was obtained from parents, including for images used in this protocol) on OpenNeuro at the end of the project. MRI data will be organised using the Brain Imaging Data Structure ( BIDS) framework.

## Study status

This project is ongoing and we are actively recruiting. Brain imaging and behavioural data has currently been collected from 48 participants.

## Conclusion

This study will quantify how bilingualism and maturational factors impact executive control and brain connectivity in the child brain. We will examine the effect of bilingualism on both domain specific (language) and domain general (EC) networks. As a result, our findings will shed light on the early effects of enriched linguistic environment on brain maturation.

## Data availability

### Underlying data

No data is associated with this article.

### Extended data

Custom written scripts that will be used to analyse each set of data are available: https://github.com/sgoksan/paed_mri_preprocessing

Archived scripts as at time of publication: http://doi.org/10.5281/zenodo.3778811 ^70^

License: MIT License
