# Supplementary material for: Early childhood bilingualism: effects on brain structure and function
Source: F1000Res. 2020 Nov 4;9:370. Originally published 2020 May 15. [Version 2] doi: 10.12688/f1000research.23216.2 (PMC7262573; doi:10.12688/f1000research.23216.2)
Supplement: Peer review history

# Peer review: Reviewer response for version 1

**DOI:** 10.5256/f1000research.25631.r63537

**Published:** 2020-05-29

**Authors:** Manuel Blesa Cábez, Paola Galdi

**Affiliations:** 1 MRC Centre for Reproductive Health, The University of Edinburgh, Edinburgh, UK

Overall the presented study is very complete and it will provide meaningful information about how bilingualism alters brain connectivity in early brain development. The presented analysis plan is appropriate to answer the questions they are addressing. The scripts (already uploaded to GitHub) will be an important resource for anyone interested to work with these (or similar) datasets, and the availability of the datasets would be an important contribution to the community, as it will be useful for other researchers investigating the mechanisms that change with bilingualism during early brain development. 

 The study is very well designed, however, I have some questions/suggestions regarding the processing of the data and the statistical analysis. The FA is generally a good biomarker for brain development, however, it is very difficult to interpret. ^1^ As the acquired multi-shell diffusion data permits it, did the authors consider calculating also NODDI maps? ^2^ In addition, did the authors consider adding some preprocessing steps like denoising or removal of Gibbs rings artifacts to the diffusion pipeline? ^3^^,^^4^

 Finally, the authors could provide more details on how they plan to control for confounding effects. One important confounder to take into account is gestational age at birth: in fact, some of the children were born between 33 and 37 weeks, and are therefore late preterm. It is well known that preterm babies have small differences in brain structure compared with term-born babies at term equivalent age, and this should be accounted for. ^5^ Then they mention that socioeconomic status will be measured on the basis of maternal education, but they don’t specify how this variable will be used in their statistical models. Another potential confounder worth considering is brain size, as it correlates to general intelligence ^6^ and it has a known effect on functional connectivity estimates (see e.g. Hänggi *et al.* ^7^). Finally, there is a large literature on sex differences in language proficiency in childhood and brain development (see Etchell *et al.* ^8^ for a systematic review). Although the authors report that there were no significant sex differences measured in normative samples for some of the scores they adopt, it would be worth clarifying the reasons for excluding this factor from the analysis.

Is the study design appropriate for the research question?

Yes

Is the rationale for, and objectives of, the study clearly described?

Yes

Are sufficient details of the methods provided to allow replication by others?

Yes

Are the datasets clearly presented in a useable and accessible format?

Yes

Reviewer Expertise:

Neonatal imaging, diffusion MRI, structural connectome, functional MRI, machine learning and statistical modelling.

We confirm that we have read this submission and believe that we have an appropriate level of expertise to confirm that it is of an acceptable scientific standard.

## References

[1] : White matter integrity, fiber count, and other fallacies: the do's and don'ts of diffusion MRI. *Neuroimage*.2013;73: 10.1016/j.neuroimage.2012.06.081 239-54 10.1016/j.neuroimage.2012.06.081 22846632

[2] : NODDI: practical in vivo neurite orientation dispersion and density imaging of the human brain. *Neuroimage*.2012;61(4) : 10.1016/j.neuroimage.2012.03.072 1000-16 10.1016/j.neuroimage.2012.03.072 22484410

[3] : Diffusion MRI noise mapping using random matrix theory. *Magn Reson Med*.76(5) : 10.1002/mrm.26059 1582-1593 10.1002/mrm.26059 26599599PMC4879661

[4] : Gibbs-ringing artifact removal based on local subvoxel-shifts. *Magn Reson Med*.76(5) : 10.1002/mrm.26054 1574-1581 10.1002/mrm.26054 26745823

[5] : Characterisation of brain volume and microstructure at term-equivalent age in infants born across the gestational age spectrum. *Neuroimage Clin*.2019;21: 10.1016/j.nicl.2018.101630 101630 10.1016/j.nicl.2018.101630 30555004PMC6411910

[6] : Meta-analysis of associations between human brain volume and intelligence differences: How strong are they and what do they mean?. *Neurosci Biobehav Rev*.2015;57: 10.1016/j.neubiorev.2015.09.017 411-32 10.1016/j.neubiorev.2015.09.017 26449760

[7] : The hypothesis of neuronal interconnectivity as a function of brain size-a general organization principle of the human connectome. *Front Hum Neurosci*.2014;8: 10.3389/fnhum.2014.00915 915 10.3389/fnhum.2014.00915 25426059PMC4227509

[8] : A systematic literature review of sex differences in childhood language and brain development. *Neuropsychologia*.114: 10.1016/j.neuropsychologia.2018.04.011 19-31 10.1016/j.neuropsychologia.2018.04.011 29654881PMC5988993

# Response

**Published:** 2020-06-09

**Authors:** Sezgi Goksan

**Affiliations:** University College London, UK

Dr Manuel Blesa Cábez and Dr Paola Galdi, 

On behalf of the authors of this manuscript, we would like to thank you both for your invaluable feedback on our project and in particular for your comments and suggestions regarding our preprocessing and statistical analysis plan. Unfortunately, due to the coronavirus pandemic, all staff employed on this project grant are being placed on furlough. Upon our return, we endeavour to respond to your comments and we intend to provide an updated version of the manuscript alongside our response.

We hope you can forgive the delay and we look forward to continuing this discussion.

Kind regards,

Dr Sezgi Goksan

# Response

**Published:** 2020-10-27

**Authors:** Sezgi Goksan

**Affiliations:** University College London, UK

Dear Dr Manuel Blesa Cábez and Dr Paola Galdi,

 Thank you for taking the time to review our manuscript. We would like to thank you both for your valuable feedback on our project and specifically, for your comments and suggestions with regards to our pre-processing and statistical analysis plan. We would like to address three points:

  

- *“The FA is generally a good biomarker for brain development, however, it is very difficult to interpret. ^1^* *As the acquired multi-shell diffusion data permits it, did the authors consider calculating also NODDI maps? ^2^”*

 

 Thank you for suggesting this additional measure. While the calculation of NODDI maps may provide complementary information regarding the underlying neurite morphology, at present we do not have any specific hypotheses with regards to how intracellular or extracellular neurite volumes would change in the bilingual brain. Similarly, we also do not report any specific hypothesis related to mean diffusivity or radial diffusivity. Given this, we have carefully considered all aforementioned measures and chosen not to include them in our statistical analysis plan. 

  

- “did the authors consider adding some pre-processing steps like denoising or removal of Gibbs rings artifacts to the diffusion pipeline?”

 

 In order to address potential participant movement artifacts within our data, we run the latest version of Eddy (a tool within FSL), which simultaneously corrects for eddy currents and gross subject movement (1-3), thus providing some denoising our data. Additional steps such as noise removal and Gibbs ringing removal are reported to be beneficial when applied prior to running EDDY (4). While such artefacts have, thus far, not been observed in the visual inspection of our diffusion data, we agree that it is important that we assess the data when finalised and investigate whether further denoising and Gibbs removal improve the quality of the pre-processing. We have acknowledged this by adding new sentences outlining this intention to our revised manuscript (see ‘Diffusion weighted data’ within the MRI data analysis section). Moreover, we are committed to updating our publicly available scripts in future in order to reflect the final pre-processing pipeline.

Added text, MRI data analysis section, *Diffusion weighted data*:

 “In addition, given our paediatric cohort, the final set of diffusion images will be carefully assessed to establish whether further denoising and removal of Gibbs ringing artefacts are required. These steps have been recommended for use in adult studies (4), and are available steps within MRTrix3 (5,6).” 

  

- “provide more details on how they plan to control for confounding effects”

 

 For this point we have specifically addressed each of the confounding variables that have been highlighted by the reviewers. Namely, (i) gestational age at birth, (ii) socio-economic status, (iii) brain size and the relationship with general intelligence and (iv) gender. 

 Firstly, we confirm that we are recording this information from each of our participants. We agree that the influence of these variables on our brain imaging data should be carefully considered and our aim is to balance these variables as best we can within each of our groups. Furthermore, we intend to examine the relationships between all of these measures in order to make an informed decision about whether to include each variable in our analysis of the brain imaging data.

  

1. Gestational age: we collect each child’s gestational age at birth via verbal report from the parent(s). At present none of our participants were born preterm, however, as mentioned we are including all children born > 33 weeks gestational age (GA) in our study. The majority of longitudinal studies investigating brain-derived measures and cognitive outcomes in preterm-born children have focussed on children born < 33 weeks GA (for example (7-10)). More recently there has been evidence suggesting that children born late preterm (i.e. 33-36 weeks GA) have higher risk of developing poor cognitive outcomes compared to their term-born peers (11). In addition, as you mention, Thompson and colleagues reported that children born late preterm had (i) larger CSF volumes and (ii) lower FA when compared to a group of term-born infants (12). However, it is not known whether such differences persist later in childhood in late preterm-born children. Therefore, we aim to assess whether the data from any preterm-born children requires individual consideration when running our final analysis. If so, we will include gestational age at birth as a confound in our analysis.
2. Socio-economic status (SES): SES impacts neurocognitive development, thereby influencing executive functions such as attention skills as well as measures of brain structure and function (13-16). In order to compare our groups fairly, we intend to match our groups for SES. As part of the Alberta Language Environment Questionnaire (17), number of years of maternal education is recorded as a measure of SES for our participants (see details of Parental Questionnaire in Methodology section). We have not specified any criteria related to SES as part of our recruitment, however in order to recruit groups from similar backgrounds we are signposting throughout our university, at local Greek schools (that provide classes at the weekend) and through inviting children and parents to mention our study to other friends at their schools. Through this approach we hope to minimise differences in SES between groups. 
3. Brain size and general intelligence: Brain size is an important factor to consider as it is related to general intelligence and brain-derived measures. This relationship has also been reported in children (18). One of our background measures, the outcome score from a Coloured Progressive Matrices (CPM) task, is included as a measure of general intelligence. We are also able to calculate a measure of brain size by extracting total cortical volume from each participant’s structural scan. This will allow us to examine the relationship between these two variables in our sample. We will include CPM scores or total cortical volume as a confound measure in our analyses of structural and functional connectivity in the brain. As such we intend to control for effects associated with general intelligence or brain size.
4. Gender: Etchell et al., argue that sex differences may impact brain development and cognitive outcomes (19). Therefore, it is our intention to balance our groups for this factor. This is an additional goal of our current, ongoing recruitment and we have thus far been able to recruit relatively gender-balanced groups. Moreover, we will examine gender-related effects and use gender as a covariate where appropriate.

 

Added text (bold), Statistical plan section:

 “ ***We will examine our data for effects related to gender, socio-economic status and gestational age at birth. We will subsequently make an informed decision regarding whether to include these factors as covariates in our connectivity analyses. ***Moreover, all analyses will include measures of **brain size (total cortical volume) or** non-verbal ability (CPM scores), working memory (scaled score for total forward and backward digit span) and age at MRI as covariates, where appropriate.”

**References:**

 1.        Andersson JLR, Sotiropoulos SN. An integrated approach to correction for off-resonance effects and subject movement in diffusion MR imaging. *NeuroImage*. 2016;125: 1063–1078. 

 2.        Andersson JLR, Graham MS, Zsoldos E, Sotiropoulos SN. Incorporating outlier detection and replacement into a non-parametric framework for movement and distortion correction of diffusion MR images. *NeuroImage*. [Online] 2016;141: 556–572. Available from: doi:10.1016/j.neuroimage.2016.06.058

 3.        Graham MS, Drobnjak I, Zhang H. Realistic simulation of artefacts in diffusion MRI for validating post-processing correction techniques. *NeuroImage*. [Online] 2016;125: 1079–1094. Available from: doi:10.1016/j.neuroimage.2015.11.006

 4.        Maximov II, Alnaes D, Westlye LT. Towards an optimised processing pipeline for diffusion magnetic resonance imaging data: Effects of artefact corrections on diffusion metrics and their age associations in UK Biobank. *Human Brain Mapping*. [Online] 2019;40(14): 4146–4162. Available from: doi:10.1002/hbm.24691

 5.        Cordero-Grande L, Christiaens D, Hutter J, Price AN, Hajnal JV. Complex diffusion-weighted image estimation via matrix recovery under general noise models. *NeuroImage*. [Online] 2019;200: 391–404. Available from: doi:10.1016/j.neuroimage.2019.06.039

 6.        Kellner E, Dhital B, Kiselev VG, Reisert M. Gibbs-ringing artifact removal based on local subvoxel-shifts. *Magnetic Resonance in Medicine*. [Online] 2016;76(5): 1574–1581. Available from: doi:10.1002/mrm.26054

 7.        Johnson S. Cognitive and behavioural outcomes following very preterm birth. *Seminars in Fetal & Neonatal Medicine*. [Online] W.B. Saunders; 2007;12(5): 363–373. Available from: doi:10.1016/j.siny.2007.05.004

 8.        Moore T, Hennessy EM, Myles J, Johnson SJ, Draper ES, Costeloe KL, et al. Neurological and developmental outcome in extremely preterm children born in England in 1995 and 2006: the EPICure studies. *BMJ*. [Online] British Medical Journal Publishing Group; 2012;345(dec04 3): e7961–e7961. Available from: doi:10.1136/bmj.e7961

 9.        Ancel P-Y, Goffinet F, Group ATE-2W, Kuhn P, Langer B, Matis J, et al. Survival and Morbidity of Preterm Children Born at 22 Through 34 Weeks’ Gestation in France in 2011: Results of the EPIPAGE-2 Cohort Study. *JAMA Pediatrics*. [Online] American Medical Association; 2015;169(3): 230–238. Available from: doi:10.1001/jamapediatrics.2014.3351

 10.      Linsell L, Johnson S, Wolke D, O’Reilly H, Morris JK, Kurinczuk JJ, et al. Cognitive trajectories from infancy to early adulthood following birth before 26 weeks of gestation: a prospective, population-based cohort study. *Archives of Disease in Childhood*. [Online] BMJ Publishing Group Ltd; 2018;103(4): 363–370. Available from: doi:10.1136/archdischild-2017-313414

 11.      Woythaler M. Neurodevelopmental outcomes of the late preterm infant. *Seminars in Fetal & Neonatal Medicine*. [Online] W.B. Saunders; 2019;24(1): 54–59. Available from: doi:10.1016/j.siny.2018.10.002

 12.      Thompson DK, Kelly CE, Chen J, Beare R, Alexander B, Seal ML, et al. Characterisation of brain volume and microstructure at term-equivalent age in infants born across the gestational age spectrum. *NeuroImage. Clinical*. [Online] Elsevier; 2019;21: 101630. Available from: doi:10.1016/j.nicl.2018.101630

 13.      Hackman DA, Farah MJ, Meaney MJ. Socioeconomic status and the brain: mechanistic insights from human and animal research. *Nature Reviews Neuroscience*. [Online] Nature Publishing Group; 2010;11(9): 651–659. Available from: doi:10.1038/nrn2897

 14.      Hackman DA, Gallop R, Evans GW, Farah MJ. Socioeconomic status and executive function: developmental trajectories and mediation. *Developmental science*. [Online] 2nd ed. John Wiley & Sons, Ltd; 2015;18(5): 686–702. Available from: doi:10.1111/desc.12246

 15.      Farah MJ. The Neuroscience of Socioeconomic Status: Correlates, Causes, and Consequences. *Neuron*. [Online] 2017;96(1): 56–71. Available from: doi:10.1016/j.neuron.2017.08.034

 16.      Lawson GM, Hook CJ, Farah MJ. A meta-analysis of the relationship between socioeconomic status and executive function performance among children. *Developmental science*. [Online] 2018;21(2). Available from: doi:10.1111/desc.12529

 17.      Paradis J. Individual differences in child English second language acquisition: Comparing child-internal and child-external factors. Hulk A, Marinis T (eds.) *Linguistic Approaches to Bilingualism*. [Online] John Benjamins; 2011;1(3): 213–237. Available from: doi:10.1075/lab.1.3.01par

 18.      Thompson DK, Matthews LG, Alexander B, Lee KJ, Kelly CE, Adamson CL, et al. Tracking regional brain growth up to age 13 in children born term and very preterm. *Nature communications*. [Online] Nature Publishing Group; 2020;11(1): 696–11. Available from: doi:10.1038/s41467-020-14334-9

 19.      Etchell A, Adhikari A, Weinberg LS, Choo AL, Garnett EO, Chow HM, et al. A systematic literature review of sex differences in childhood language and brain development. *Neuropsychologia*. [Online] 2018;114: 19–31. Available from: doi:10.1016/j.neuropsychologia.2018.04.011

# Peer review: Reviewer response for version 1

**DOI:** 10.5256/f1000research.25631.r63542

**Published:** 2020-05-27

**Authors:** Christos Pliatsikas

**Affiliations:** 1 School of Psychology and Clinical Language Sciences, University of Reading, Reading, UK

This manuscript present the research protocol of a well thought through and designed ongoing study on the effects of bilingualism on the brain structure and function of children. This study fills an important gap in the young but expanding literature on the effects of bilingualism on cognition and the brain, by expanding it to a severely under-researched age-range (young children)- crucially, the study is administering comprehensive behavioural and MRI batteries which will help uncover whether and how brain differences between bilingual and monolingual children translate to differences in behaviour. 

 I found the rationale and objectives of the study clearly described, and the study design is very appropriate, although the use of the entire behavioural battery could have also been addressed with appropriate hypotheses, in order to further strengthen the study. In terms of methods, all three main testing components (demographic and language backgrounds, behavioural experiments tapping language and executive functions and the MRI battery), are appropriate, contemporary, and described in full detail allowing for replication of the study. The adaptations of the MRI data analyses to make them appropriate for a paediatric sample is of particular importance here. Finally, with respect to data analysis, it is not clear to me whether the authors were planning to look at grey matter measures (esp. since their T1 images have sufficient resolution) with methods included in FSL, such as whole-brain VBM and subcortical vertex analysis (FIRST). I would strongly advise that, as it will result in a much needed multi-modal investigation of the developing bilingual brain (e.g. see https://psyarxiv.com/kjq6m for a preprint of our recent work looking at both grey and white matter structure of the developing brain, but not brain function at rest (Pliatsikas *et al.*, 2020 ^1^). In all, this is a well-designed study and its outputs will be more than welcome in the field.

Is the study design appropriate for the research question?

Yes

Is the rationale for, and objectives of, the study clearly described?

Yes

Are sufficient details of the methods provided to allow replication by others?

Yes

Are the datasets clearly presented in a useable and accessible format?

Not applicable

Reviewer Expertise:

Bilingualism, experience-dependent neuroplasticity

I confirm that I have read this submission and believe that I have an appropriate level of expertise to confirm that it is of an acceptable scientific standard.

## References

[1] : The effect of bilingualism on brain development from early childhood to young adulthood.2020; 10.31234/osf.io/kjq6m 10.31234/osf.io/kjq6m PMC747397232691216

# Response

**Published:** 2020-06-09

**Authors:** Sezgi Goksan

**Affiliations:** University College London, UK

Dr Christos Pliatsikas, 

 On behalf of the authors of this manuscript, we would like to thank you for your invaluable feedback on our project. Unfortunately, due to the coronavirus pandemic, all staff employed on this project grant are being placed on furlough. Upon our return, we endeavour to respond to your comments and we intend to provide an updated version of the manuscript alongside our response.

 We hope you can forgive the delay and we look forward to continuing this discussion.

 Kind regards,

 Dr Sezgi Goksan.

# Response

**Published:** 2020-10-27

**Authors:** Sezgi Goksan

**Affiliations:** University College London, UK

Dear Dr Christos Pliatsikas, 

 Thank you for taking the time to review our manuscript and for your valuable feedback on our project. We would like to address two of your comments:

  

- *“I found the rationale and objectives of the study clearly described, and the study design is very appropriate, although the use of the entire behavioural battery could have also been addressed with appropriate hypotheses, in order to further strengthen the study.”*

 

 With regards to reporting specific hypotheses related to our executive control tests, given the contradictory evidence regarding a ‘bilingual advantage’ in executive control in adult studies (for example see (1,2) for reviews in favour and (3-5) for reviews arguing against), and the few studies investigating EC in childhood (see reviews from (6,7)), there is insufficient evidence for us to formulate a specific hypothesis. Instead, we intend to explore the behavioural data alongside the brain imaging data. In doing so, we hope to meaningfully contribute this debate. Furthermore, for our background measures, namely the tests of non-verbal fluid intelligence and verbal working memory, we have included new sentences elaborating on the use of these measures in the current literature (see ‘ *Background measures’* within the Methodology section in our revised manuscript).

Added text, Methodology section, *Background measures*:

 “Fluid intelligence, working memory and controlled attention are related yet separable constructs (8-10). We have therefore chosen to collect measures of non-verbal fluid intelligence and verbal working memory as background variables, as in previous studies that have investigated executive control skills in bilingual children and adults (11-15). Based on Engle et al.’s theory, individual differences in working memory capacity and general intelligence will impact a child’s innate ability to control their attention (9). Moreover, it has been observed that greater resting-state connectivity of the precuneus/posterior cingulate with other regions of the default mode network, is positively correlated with working memory performance in adults (16). Therefore, we aim to account for individual variability related to measures of executive control by including scores for working memory and general intelligence as confounding factors in our connectivity analyses.”

  

- *“with respect to data analysis, it is not clear to me whether the authors were planning to look at grey matter measures”*. 

 

 In this protocol we have chosen to focus specifically on measures of structural and functional connectivity in children, as this is the novel aspect of our study. We have therefore not described plans within for analysis of the cortical structures. However, given that we are collecting high-resolution structural images, this is certainly an avenue that can be explored further in the future as a complementary analysis project in which we would aim to replicate and extend results in the existing published literature (17,18). We are committed to sharing further analysis plans via additional scripts for the pre-processing and analysis of structural data on our github page: https://github.com/sgoksan/paed_mri_preprocessing

**References**

 1.        Adesope OO, Lavin T, Thompson T, Ungerleider C. A Systematic Review and Meta-Analysis of the Cognitive Correlates of Bilingualism:. *Review of Educational Research*. [Online] SAGE PublicationsSage CA: Los Angeles, CA; 2010;80(2): 207–245. Available from: doi:10.3102/0034654310368803

 2.        Bialystok E. Reshaping the mind: the benefits of bilingualism. *Canadian journal of experimental psychology = Revue canadienne de psychologie experimentale*. [Online] 2011;65(4): 229–235. Available from: doi:10.1037/a0025406

 3.        Hilchey MD, Klein RM. Are there bilingual advantages on nonlinguistic interference tasks? Implications for the plasticity of executive control processes. *Psychonomic Bulletin & Review*. [Online] Springer-Verlag; 2011;18(4): 625–658. Available from: doi:10.3758/s13423-011-0116-7

 4.        Lehtonen M, Soveri A, Laine A, Järvenpää J, de Bruin A, Antfolk J. Is bilingualism associated with enhanced executive functioning in adults? A meta-analytic review. *Psychological bulletin*. [Online] 2018;144(4): 394–425. Available from: doi:10.1037/bul0000142

 5.        Donnelly S, Brooks PJ, Homer BD. Is there a bilingual advantage on interference-control tasks? A multiverse meta-analysis of global reaction time and interference cost. *Psychonomic Bulletin & Review*. [Online] Springer US; 2019;26(4): 1122–1147. Available from: doi:10.3758/s13423-019-01567-z

 6.        Barac R, Bialystok E, Castro DC, Sanchez M. The Cognitive Development of Young Dual Language Learners: A Critical Review. *Early childhood research quarterly*. [Online] 2014;29(4): 699–714. Available from: doi:10.1016/j.ecresq.2014.02.003

 7.        Ware AT, Kirkovski M, Lum JAG. Meta-Analysis Reveals a Bilingual Advantage That Is Dependent on Task and Age. *Frontiers in Psychology*. [Online] Frontiers Media SA; 2020;11: 2076. Available from: doi:10.3389/fpsyg.2020.01458

 8.        Baddeley AD, Hitch G. Working Memory. *Psychology of Learning and Motivation*. [Online] Academic Press; 1974;8: 47–89. Available from: doi:10.1016/S0079-7421(08)60452-1

 9.        Engle RW, Kane MJ, Tuholski SW. Individual differences in working memory capacity and what they tell us about controlled attention, general fluid intelligence, and functions of the prefrontal cortex. 1999. Available from: doi:10.1017/CBO9781139174909.007

 10.      Miyake A, Friedman NP. The Nature and Organization of Individual Differences in Executive Functions: Four General Conclusions. *Current Directions in Psychological Science*. [Online] SAGE PublicationsSage CA: Los Angeles, CA; 2012;21(1): 8–14. Available from: doi:10.1177/0963721411429458

 11.      Namazi M, Thordardottir E. A working memory, not bilingual advantage, in controlled attention. *International Journal of Bilingual Education and Bilingualism*. [Online] 3rd ed. Taylor & Francis Group; 2010;13(5): 597–616. Available from: doi:10.1080/13670050.2010.488288

 12.      Bonifacci P, Giombini L, Bellocchi S, Contento S. Speed of processing, anticipation, inhibition and working memory in bilinguals. *Developmental science*. [Online] John Wiley & Sons, Ltd; 2011;14(2): 256–269. Available from: doi:10.1111/j.1467-7687.2010.00974.x

 13.      Engel de Abreu PMJ. Working memory in multilingual children: is there a bilingual effect? *Memory (Hove, England)*. [Online] 2nd ed. Taylor & Francis Group; 2011;19(5): 529–537. Available from: doi:10.1080/09658211.2011.590504

 14.      D'Souza AA, Moradzadeh L, Wiseheart M. Musical training, bilingualism, and executive function: working memory and inhibitory control. *Cognitive research: principles and implications*. [Online] 2nd ed. SpringerOpen; 2018;3(1): 11–18. Available from: doi:10.1186/s41235-018-0095-6

 15.      Antón E, Carreiras M, Duñabeitia JA. The impact of bilingualism on executive functions and working memory in young adults. Athanasopoulos P (ed.) *Public Library of Science ONE*. [Online] Public Library of Science; 2019;14(2): e0206770. Available from: doi:10.1371/journal.pone.0206770

 16.      Sala-Llonch R, Peña-Gómez C, Arenaza-Urquijo EM, Vidal-Piñeiro D, Bargalló N, Junqué C, et al. Brain connectivity during resting state and subsequent working memory task predicts behavioural performance. *Cortex; a journal devoted to the study of the nervous system and behavior*. [Online] 2012;48(9): 1187–1196. Available from: doi:10.1016/j.cortex.2011.07.006

 17.      Archila-Suerte P, Woods EA, Chiarello C, Hernandez AE. Neuroanatomical profiles of bilingual children. *Developmental science*. [Online] 2018;21(5): e12654. Available from: doi:10.1111/desc.12654

 18.      Thieba C, Long X, Dewey D, Lebel C. Young children in different linguistic environments: A multimodal neuroimaging study of the inferior frontal gyrus. *Brain and cognition*. [Online] 2019;134: 71–79. Available from: doi:10.1016/j.bandc.2018.05.009
